# Peripheral Nerve Stimulation for the Treatment of Phantom Limb Pain: A Case Series

**DOI:** 10.1155/2023/1558183

**Published:** 2023-03-06

**Authors:** Robert Pagan-Rosado, Brandon J. Smith, Fantley C. Smither, Matthew J. Pingree, Ryan S. D'Souza

**Affiliations:** ^1^Department of Physical Medicine and Rehabilitation, Mayo Clinic, Rochester, MN, USA; ^2^Department of Anesthesiology and Perioperative Medicine, Division of Pain Medicine, Mayo Clinic Hospital, Rochester, MN, USA

## Abstract

This case series aims to highlight the efficacy of peripheral nerve stimulation (PNS) in the treatment of phantom limb pain, as well as provide an alternative method for the treatment of this pain syndrome. In this report, we describe three amputee patients with severe phantom limb pain who obtained substantial analgesia and improvement in physical functionality after implantation of a temporary PNS device. Future studies should assess predictors of successful response or poor response to PNS therapy, such as mental health, environmental stressors, coping skills, and procedural factors. These factors may facilitate an individualized approach for each patient to ensure appropriate candidacy for PNS and better prognosis. Considering that patients in our cohort did not achieve long-lasting benefit after removal of temporary PNS, future research should assess if patients with phantom limb pain would benefit from permanent PNS, rather than temporary PNS.

## 1. Introduction

Nearly 200,000 extremity amputations are performed annually in the United States [1]. Despite advances in the field of pain medicine, nearly 70% of post-amputee patients experience long-term chronic pain and discomfort following the amputation [2]. Prolonged and uncontrolled pain is associated with multiple negative consequences including poor quality of life and functionality. Post-amputation pain is generally categorized as either phantom limb pain (PLP) and/or residual limb pain (RLP). PLP is pain originating in the lost limb that is no longer present to generate sensory input and is believed to have both central and peripheral components. There are a variety of descriptors for PLP including burning, stabbing, itching, or muscular contractions and spasms [3]. First-line treatment for PLP typically begins with evaluating prosthetic fit and wound healing and also includes mirror therapy and desensitization. Commonly utilized pharmacologic agents include gabapentinoids and selective norepinephrine reuptake inhibitors. Despite these measures, many patients with PLP fail to achieve adequate analgesia [3], which has resulted in growing interest in the use of neuromodulation to address this pain. Specifically, peripheral nerve stimulation (PNS) has recently emerged as a neuromodulation intervention that can be utilized to treat refractory manifestations of chronic pain, including post-amputation pain [4, 5]. This case series aims to highlight the efficacy of PNS in three patients with phantom limb pain from a single tertiary academic center as well as propose a treatment algorithm for phantom limb pain that incorporates the use of PNS.

## 2. Patient Information, Therapeutic Intervention, and Outcomes

### 2.1. Case 1

A 68-year-old female with a history of left transhumeral amputation, end-stage renal disease on hemodialysis, hypertension, type 2 diabetes mellitus, and anxiety presented to the pain clinic for phantom limb pain. Her left transhumeral amputation was due to a planned surgical resection due to necrotizing fasciitis two years ago.

Her main complaint was severe phantom limb pain based around the area where her thumb, dorsal hand, and wrist were previously located. She reported constant shooting and burning pain with intermittent “lightning bolt” sensations. She also described residual nociceptive limb pain which was less intense compared to the phantom limb pain. She had previously trialed gabapentin 100 mg twice a day, nortriptyline 10 mg daily, 5 mg oxycodone four times daily, lidocaine patches, ice application, and mirror therapy without success.

**Figure 1:**
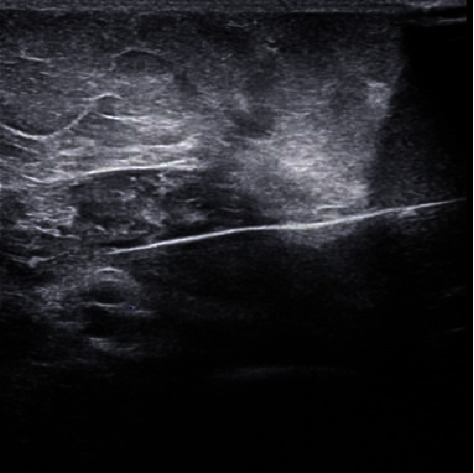
Transverse sonographic view of the median nerve proximal to the left antecubital fossa, with an in-plane stimulator needle superior to the median nerve. Due to poor quality, the sonographic images of the other two cases were not included.

A median nerve neuroma was identified during examination with ultrasound, and thus, the decision was made to perform PNS of the left median nerve for pain relief. A diagnostic block with 2% lidocaine at the left median nerve resulted in 80% pain relief. A temporary stimulator (SPRINT, SPR Therapeutics, Cleveland, OH, USA) was implanted under ultrasound guidance in close proximity to the left median nerve. A Sonosite linear ultrasound transducer (15-6 MHz) was used to identify the left median nerve (neuroma). A short-axis, in-plane approach was used to place the stimulating needle in close proximity to the neuroma (Figure 1). The introducer needle was advanced to near the left median nerve (neuroma) and stimulation was carried out with the patient stating that she was feeling paresthesia in the phantom limb (thumb and index finger). After the stimulating needle exhibited optimal spread of coverage, a PNS lead was advanced through the stimulating needle. The external portion of the lead was secured to the skin with Dermabond (Ethicon, Inc., Somerville, NJ) and Tegaderm (3M, Inc., Saint Paul, MN). At the 2-week follow-up after implantation, the patient reported a 40% improvement in both pain and physical functionality, with a subsequent 60% improvement in both outcomes at the 6-weekfollow-up period. Further, she reported substantially lower frequency of pain flares and great satisfaction with PNS. After completion of the 60-day PNS treatment and removal of the device, her pain returned to its baseline within 3 months. No complications were reported during or after the procedure.

### 2.2. Case 2

A 77-year-old female has a history of hypertension, diabetes, chronic kidney disease, depression, and a high-grade spindle cell sarcoma that resulted in a left transfemoral amputation. She reported phantom limb pain diffusely around the area of the left foot. She had previously tried gabapentin, duloxetine, tramadol, intra-articular hip steroid injections, and mirror therapy without any significant relief. The pain was described as severe 10/10 pain exacerbated by weather changes, and intermittent radiation to the residual stump.

She underwent a left femoral and sciatic nerve ultrasound-guided diagnostic block with 2% lidocaine which provided significant pain relief (100%) for 4–6 hours. Two months later, she underwent a temporary PNS device placement targeting the left femoral and sciatic nerves. A Sonosite linear ultrasound transducer (15-6 MHz) was used to identify the left femoral and sciatic nerve at the inguinal and subgluteal region, respectively. A short-axis, in-plane approach was used to place the stimulating needle in close proximity to the nerves. The subcutaneous tissue layers were identified, and local anesthetic was infiltrated in the subcutaneous tissue. The introducer needle was first advanced to near the left femoral nerve and stimulation was carried out with the patient stating that she was feeling paresthesia in the left femoral nerve distribution. We then inserted the lead and re-performed stimulation and then styleted the lead. We repeated these same steps for the left sciatic nerve. After the stimulating needle exhibited optimal spread of coverage, a PNS lead was advanced through the stimulating needle. The external portion of the lead was secured to the skin with Dermabond (Ethicon, Inc., Somerville, NJ) and Tegaderm (3M, Inc., Saint Paul, MN).

At the 2-week follow-up visit, the patient reported a 100% improvement for both pain intensity and physical functionality, with a sustained 100% improvement in both measures at the 6-week follow-up visit. She reported lower frequency of pain flares and greater satisfaction with PNS therapy compared to baseline. After severe recurrence of pain was noted at day 45, ultrasound evaluation revealed migration of the PNS lead. The PNS settings were re-programmed, and efficacy was achieved again with 75% improvement in pain intensity. After removal of the PNS device 60 days post-implant, she had a relapse of phantom limb pain in 2 months that was 50% less intense than pre-implant pain intensity. No other complications were reported.

### 2.3. Case 3

A 16-year-old male reported a history of chronic left lower extremity pain in the setting of refractory erythromelalgia and left transtibial amputation secondary to severe pain and recurrent infection. After amputation, he began experiencing severe phantom limb pain at the left foot about three weeks after amputation. A diagnostic nerve block at the sciatic nerve provided 80% pain relief, and therefore, a temporary PNS stimulator was implanted under ultrasound guidance to stimulate the sciatic nerve. A Sonosite linear ultrasound transducer (15-6 MHz) was used to identify the left sciatic nerve at the subgluteal region. A short-axis, in-plane approach was used to place the stimulating needle in close proximity to the nerve. The subcutaneous tissue layers were identified, and local anesthetic was infiltrated in the subcutaneous tissue. The introducer needle was advanced to near the left sciatic nerve and stimulation was carried out with the patient stating that he was feeling paresthesia in the left sciatic nerve distribution. We then inserted the lead and repeated stimulation and then styleted the lead. After the stimulating needle exhibited optimal spread of coverage, a PNS lead was advanced through the stimulating needle to the optimal site of stimulation. The external portion of the lead was secured to the skin with Dermabond (Ethicon, Inc., Somerville, NJ) and Tegaderm (3M, Inc., Saint Paul, MN).

At the 2-week follow-up visit, the patient reported pain intensity was 2/10 compared to 10/10 at baseline. At the 6-week follow-up visit, pain intensity was 5/10. He reported less frequent pain flares and great satisfaction. After removal of his PNS device at 60 days, his pain intensity subsequently increased to 10/10 within one month following removal. No complications were reported.

## 3. Discussion

We report a case series of post-amputee patients with severe phantom limb pain who obtained substantial analgesia and improvement in physical functionality after implantation of a temporary PNS device. Reduced frequency of pain flares, good patient satisfaction, and no adverse events were reported. However, after removal of the temporary PNS device, pain intensity worsened in all patients and regressed completely back to pre-implant baseline levels in two patients. This small case series highlights that temporary PNS provides substantial short-term pain relief and improved physical functionality, although this therapeutic effect is temporary and does not extend long-term after device removal.

The mechanism in which patients experience PLP is not entirely delineated but likely has both central and peripheral components. Central components include somatosensory cortical reorganization of the area representing the amputated limb, as well as spinal reorganization in the dorsal horns after deafferentation from a peripheral nerve [6]. Peripheral components such as increased ectopic nociceptive afferent inputs, axonal nerve inflammation, and regenerative sprouting may also contribute to PLP [7]. PNS is also believed to have both peripheral and central mechanisms contributing, which would lay the foundation for a plausible mechanism of PLP improvements with PNS. Central alterations with PNS include the activation of afferent non-nociceptive fibers which alter dorsal horn interneurons that are needed in the processing of nociceptive stimuli. PNS may also modulate wide dynamic range neurons in the dorsal horns [8] and can act peripherally by altering local micro-environments of nociceptive afferent fibers and reducing ectopic discharges [9]. PNS likely results in improvements in PLP by altering both the central and peripheral mechanism contributing to PLP.

Our results are concordant with the literature, which highlights efficacy of PNS in treatment of post-amputation pain [4, 5, 10]. Our data are also consistent with superior analgesia and clinical outcomes from PNS treatment of both acute and chronic pain syndromes in the back, upper extremity, lower extremity, and head [4, 11]. However, PNS studies utilizing temporary PNS lead placement highlight persistent pain relief up to a year after removal of the PNS device. Our small case series contradicts this finding because all patients in our series reported significant worsening of pain intensity within a month after PNS device removal. Potential explanations for this include comorbid psychiatric and medical comorbidities in all three patients, which may have amplified their pain intensity and affected their response to PNS. Moreover, prior literature on PNS for post-amputation pain excluded certain patients, such as those with diabetes, bleeding disorders, autoimmune disorders, and depression, which may explain the differences in outcomes between our case series and the results of prior studies [4, 5]. Interestingly, patient #3 in this series had a history of erythromelalgia, which may be an additional factor leading to treatment-resistant and refractory pain, given its interplay with the peripheral vasculature and peripheral nervous system.

Additional clinical studies are needed to further assess the efficacy of PNS in the treatment of phantom limb pain. The current literature suggests that PNS therapy may provide clinically significant pain relief and improvement in the quality of life of patients with chronic neuropathic post-amputation pain, which usually includes both residual limb pain and phantom limb pain [4, 5, 10, 12].

When evaluating post-amputee patients who are experiencing phantom limb pain, the first step should be to assess prosthetic fit and ensure proper wound healing if amputation occurred recently. If none of these are sources of pain, referral to a physical therapist would be warranted to work on multiple exercises and pain management modalities. Pharmacologic treatment with oral gabapentinoids, selective norepinephrine reuptake inhibitors (SNRIs), or tricyclic antidepressants (TCAs), followed with a formal biopsychologic evaluation, should be the next step in cases where pain is refractory to rehabilitation and alternative therapies. Neuromodulation techniques such as PNS and spinal cord stimulation may be considered for those patients who do not obtain benefit from conservative and pharmacologic treatment. Additionally, other procedural interventions such as dorsal root entry zone ablation, peripheral nerve blocks, and radiofrequency ablations can also be considered. In some cases, surgical intervention may be necessary if none of the aforementioned interventions improve the patients' pain and quality of life.

Future studies should assess predictors of successful response or poor response to PNS therapy, such as mental health, environmental stressors, coping skills, and procedural factors. These factors may facilitate an individualized approach for each patient to ensure appropriate candidacy for PNS and better prognosis. Considering that patients in our cohort did not achieve long-lasting benefits after removal of temporary PNS, future research should assess if patients with phantom limb pain would benefit from permanent PNS, rather than temporary PNS.

## References

[1] Ziegler-Graham K., MacKenzie E. J., Ephraim P. L., Travison T. G., Brookmeyer R. (2008). Estimating the prevalence of limb loss in the United States: 2005 to 2050. *Archives of Physical Medicine and Rehabilitation*.

[2] Ehde D. M., Czerniecki J. M., Smith D. G. (2000). Chronic phantom sensations, phantom pain, residual limb pain, and other regional pain after lower limb amputation. *Archives of Physical Medicine and Rehabilitation*.

[3] McCormick Z., Chang-Chien G., Marshall B., Huang M., Harden R. N. (2014). Phantom limb pain: a systematic neuroanatomical-based review of pharmacologic treatment. *Pain Medicine*.

[4] Gilmore C., Ilfeld B., Rosenow J. (2019). Percutaneous peripheral nerve stimulation for the treatment of chronic neuropathic postamputation pain: a multicenter, randomized, placebo-controlled trial. *Regional Anesthesia and Pain Medicine*.

[5] Albright-Trainer B., Phan T., Trainer R. J. (2021). Peripheral nerve stimulation for the management of acute and subacute post-amputation pain: a randomized, controlled feasibility trial. *Pain Management*.

[6] Kew J. J., Ridding M. C., Rothwell J. C. (1994). Reorganization of cortical blood flow and transcranial magnetic stimulation maps in human subjects after upper limb amputation. *Journal of Neurophysiology*.

[7] Hsu E., Cohen S. P. (2013). Postamputation pain: epidemiology, mechanisms, and treatment. *Journal of Pain Research*.

[8] Papuć E., Rejdak K. (2013). The role of neurostimulation in the treatment of neuropathic pain. *Annals of Agricultural and Environmental Medicine: AAEM*.

[9] Strand N. H., D’Souza R., Wie C. (2021). Mechanism of action of peripheral nerve stimulation. *Current Pain and Headache Reports*.

[10] Cohen S. P., Gilmore C. A., Rauck R. L. (2019). Percutaneous peripheral nerve stimulation for the treatment of chronic pain following amputation. *Military Medicine*.

[11] Wilson R. D., Gunzler D. D., Bennett M. E., Chae J. (2014). Peripheral nerve stimulation compared with usual care for pain relief of hemiplegic shoulder pain: a randomized controlled trial. *American Journal of Physical Medicine and Rehabilitation*.

[12] Gilmore C. A., Kapural L., McGee M. J., Boggs J. W. (2018). Percutaneous peripheral nerve stimulation (PNS) for the treatment of chronic low back pain provides sustained relief. *Neuromodulation*.

